# Thoracic adipose tissue contributes to severe virus infection of the lung

**DOI:** 10.1038/s41366-023-01362-w

**Published:** 2023-08-16

**Authors:** Franziska Hornung, Luise Schulz, Nilay Köse-Vogel, Antje Häder, Jana Grießhammer, Daniel Wittschieber, Angelina Autsch, Christina Ehrhardt, Gita Mall, Bettina Löffler, Stefanie Deinhardt-Emmer

**Affiliations:** 1 Institute of Medical Microbiology, Jena University Hospital, Am Klinikum 1, Jena, Germany; 2 Institute of Forensic Medicine, Jena University Hospital, Am Klinikum 1, Jena, Germany; 3 Institute of Forensic Medicine, University Hospital Bonn, University of Bonn, Stiftsplatz 12, 53111 Bonn, Germany; 4 Section of Experimental Virology, Institute of Medical Microbiology, Center for Molecular Biomedicine (CMB), Jena University Hospital, Hans-Knoell-Straße 2, Jena, Germany; 5 Leibniz Institute of Photonic Technology-Member of the Research Alliance “Leibniz Health Technologies”, Albert-Einstein-Straße 9, Jena, Germany

**Keywords:** Risk factors, Microbiology

## Abstract

**Objective:**

Obesity is an independent risk factor for severe influenza virus and COVID-19 infections. There might be an interplay between adipose tissue and respiratory pathogens, although the mechanism is unknown. Proinflammatory factors secreted by the adipose tissue are often discussed to serve as indirect contributor to virus infection. However, the direct potential of adipose tissue to serve as a viral niche has not yet been investigated.

**Methods:**

Two murine obesity models (DIO and *ob/ob*) were infected with influenza A virus (IAV) and monitored for 3 weeks. p.i. Lung and adipose tissue were harvested, and the viral load was analysed. Direct replication of IAV in vitro was investigated in human derived primary adipocytes and macrophages. The indirect impact of the secretory products of adipocytes during infection was analysed in a co-culture system with lung fibroblasts. Moreover, lung and adipose tissue was harvested from deceased patients infected with SARS-CoV-2 omicron variant. Additionally, replication of SARS-CoV-2 alpha, delta, and omicron variants was investigated in vitro in adipocytes and macrophages.

**Results:**

Both murine obesity models presented high IAV titers compared to non-obese mice. Interestingly, adipose tissue adjacent to the lungs was a focal point for influenza virus replication in mice. We further detected IAV replication and antiviral response in human adipocytes. Co-cultivation of adipocytes and lung fibroblasts led to increased IL-8 concentration during infection. Though we observed SARS-CoV-2 in the thoracic adipose tissue of COVID-19 patients, no active replication was found in adipocytes in vitro. However, SARS-CoV-2 was detected in the macrophages and this finding was associated with increased inflammation.

**Conclusions:**

Our study revealed that thoracic adipose tissue contributes to respiratory virus infection. Besides indirect induction of proinflammatory factors during infection, adipocytes and macrophages within the tissue can directly support viral replication.

## Introduction

The continuous emergence of novel respiratory viruses leads to recurring outbreaks of infections even in the 21st century [1, 2]. In 2009, a new influenza A virus (IAV) strain caused an influenza pandemic and recently, severe acute respiratory syndrome coronavirus type 2 (SARS-CoV-2) created a global health emergency [2, 3]. Epidemiological profiles of these viral infections have disclosed heretofore unknown potential risk factors for severe illness. In addition to crucial comorbidities such as age and immunosuppression, obesity is apparently another independent risk factor for severe viral respiratory infection [4, 5]. Recently, epidemiological data revealed associations among coronavirus disease 2019 (COVID-19), high body mass index (BMI), and mortality [5].

As of 2016, there were over 1.9 billion adults with overweight worldwide and the number of persons with obesity has tripled since 1975 [6]. In general, obesity is a condition characterized by an abnormal and excessive increase in adipose tissue mass beyond physical requirements [7, 8]. The World Health Organization (WHO) defines obesity and overweight as excessive fat accumulation leading to elevated morbidity rates for various health problems [6]. Adipose tissue stores metabolic energy and also functions as an endocrine gland secreting various factors that cause low-grade inflammation [6]. Moreover, it expresses macrophage-related proinflammatory genes [9]. Sun et al. proposed that adipocyte death, chemotactic regulation, hypoxia, and fatty acid flux are key potential initiators of macrophage immigration in adipose tissue [10].

The aim of the present study was to investigate the role of adipose tissue in the context of lung infections. Interestingly, our results demonstrated that adipose tissue is a site of replication for influenza viruses but not for SARS-CoV-2. However, adipocytes and macrophages contribute to the inflammation associated with these infections.

## Methods

### Viral strains and plaque assay

H1N1 influenza strain Influenza A/Jena/5852/09 (variant) HA-G222 (pdmH1N1) was isolated as previously described and used for in vivo infection [11]. All in vitro infections were performed using the H1N1 influenza A virus/Puerto Rico/8/34 (IAV/PR8) strain. IAV strains were propagated in Madin-Darby canine kidney (MDCK) cells kindly provided by the Section of Experimental Virology, Institute of Medical Microbiology and cultivated in Eagle’s minimum essential medium (EMEM) (Lonza Group, Allendale, NJ, USA) supplemented with 10% (v/v) fetal bovine serum (FBS; PAN-Biotech GmbH, Aidenbach, Germany) and 1% (w/v) penicillin/streptomycin (Lonza Group).

SARS-CoV-2 variants SARS-CoV-2/hu/Germany/Jena-vi005588/2020 (alpha variant, GenBank MW633324.1) and SARS-CoV-2/human/DEU/vi0114749/2021 (delta variant, GenBank ON650061.1) were isolated from respiratory specimens of patients at the Jena University Hospital (ethics approval No. 2018-1263) as previously described [12]. SARS-CoV-2 omicron variant (human, 2021, Germany, B.1.1.529) was acquired from the European Virus Archive Global (EvaG; Marseille, France) and propagated in Vero-ACE2 cells (InvivoGen, San Diego, CA, USA) cultivated in DMEM supplemented with 10% (v/v) FBS, 1% (w/v) penicillin/streptomycin, and 125 µg/mL Hygromycin B Gold (InvivoGen). Standard plaque assays were performed either on MDCK (IAV) or Vero-76 (SARS-CoV-2) cells as previously described [12, 13]. Both used cell lines were tested negative for mycoplasma.

### Mouse models and infection

In vivo experiments were approved by the Office for Consumer Protection of Thuringia (TV-Nr: 02-018/16). All animal studies were non-randomized and no blinding was done.

The diet-induced-obesity (DIO) model, female BALB/cJRj wild type (WT) mice age 5 weeks were procured from Janvier Labs (Le Genest-Saint-Isle, France). For 12 weeks, they were fed a high-fat diet (HFD) (ssniff Spezialdiäten GmbH, Soest, Germany) consisting of 60 kJ% fat, 20 kJ% protein, and 20 kJ% carbohydrates. Equation 1 shows the formula used to confirm obesity: Obese ≥ mean (body weight NFD) + 3 × SD (body weight NFD). Mice that did not meet the criterion for the high-fat diet (HFD) were excluded from the analysis. The sample size for uninfected control mice consisted of *n* = 3 for the non-obese group and *n* = 6 for the HFD group at each time point. For infected mice, the sample size was scheduled as *n* = 20 for the non-obese group and *n* = 40 for the HFD group at each time point.

Female *ob/ob* C57Bl/6 mice characterized by global leptin KO (B6.V-Lep *ob/ob* JRj; age 8 weeks; Janvier Labs) and lean heterozygote control mice (B6.V-Lep ob/+ JRj témoin; age 8 weeks; Janvier Labs) were used as secondary murine obesity models. The sample size for uninfected control mice was *n* = 5 for both genotypes. For infected mice, the sample size was *n* = 10 for the non-obese group and *n* = 10 for the HFD group at each time point.

Mice were anesthetized with 2% isoflurane and intranasally infected with 10^6^ plaque forming units (PFU) HA-G222-mpJena/5258 strain diluted in 0.9% (w/v) NaCl. Body weight and overall burden score were measured daily for 21 days (Table S1). Active viral particles and cytokines were analyzed in the right inferior lung lobes. Therefore, tissue was homogenized in the appropriate amount of cell culture medium EMEM (Lonza Group) to adjust each specimen to its weight in mg. After homogenization, samples were centrifuged for 10 min at 4000 rpm, and cell-free supernatants were stored at −80 °C until assay performance. The number of infectious virus particles in PFU per ml was determined by standard plaque assay. To analyze gene expression in the extracted abdominal and thoracic adipose tissue, the samples were homogenized in RLT buffer prior to RNA extraction.

### COVID-19 patient autopsy and tissue processing

Autopsies were conducted at the very early postmortem stage by two experienced forensic pathologists. The general study protocol was approved by the local ethics board under registration No. 2020-1773 and included recording relevant metadata and validating native and nonfixed tissue and organ samples [14]. Pericardium, epicardium, mediastinum/periaortic, and mesenterium (fatty tissues) as well as right superior lobes, right middle lobes, right inferior lobes, left superior lobes, and left inferior lobes (lungs) were collected from five COVID-19 patients (Table S2).

### Human primary adipocyte differentiation and infection

Human white preadipocytes and their corresponding incubation media were obtained from PromoCell (Heidelberg, Germany) and negative tested for mycoplasma. Differentiation was conducted according to the manufacturer’s instructions (Fig. S1). Mature adipocytes were infected with IAV/PR8 at a multiplicity of infection (MOI) of 1. Cells were incubated with viral dilutions prepared in Dulbecco’s phosphate-buffered saline (DPBS; Thermo Fisher Scientific, Waltham, MA, USA), supplemented with 0.2% (v/v) bovine serum albumin (BSA; Carl Roth GmbH, Karlsruhe, Germany), 1 mM MgCl_2_, and 0.9 mM CaCl_2_ at 37 °C and 5% CO_2_ for 30 followed by a wash step with DPBS. Afterwards, cells were incubated in adipocyte nutrition medium supplemented with 1 mM MgCl_2_, 0.9 mM CaCl_2_, and 30 ng *L*-1-tosylamido-2-phenylethyl chloromethyl ketone (TPCK)-treated trypsin (Thermo Fisher Scientific) for 1 day and 3 days. Infection with the SARS-CoV-2 alpha, delta, and omicron variants was performed at MOI1. Cells were infected with virus diluted in adipocyte nutrition medium supplemented with 10% (v/v) FBS for 60 min. After one washing step cells were cultured in nutrition medium for 1 day and 3 days.

### Detection of sialic acid residues as cell receptors for IAV hemagglutinin

To assess the abundance of sialic acid (SA) residues attached to galactose in differentiated human primary adipocytes, we employed specific, biotinylated lectins. Sambucus Nigra Lectin (SNA, VEC-B-1305-2, Biozol, Eching Germany) was used to detect *α*-2,6-linked SA, while Maackia Amurensis Lectin II (MAL II, VEC-B-1265-1, Biozol) to detect *α*-2,3-linked SAs. Differentiated adipocytes were seeded on coverslips in 24-well plates and fixed with 4% PFA for 30 min at 37 °C, 5% CO_2_. Following fixation, cells were blocked with 3% BSA for 30 min at RT and then incubated with SNA (1:100) or MAL II (1:100). After three washing steps, cells were incubated with Streptavidin−Cy3™ (1:100, S6402, Sigma Aldrich, St. Louis, MO, USA) and Alexa Fluor™ 488 Phalloidin (1:400; A12379, invitrogen, Carlsbad, CA, USA) to visualize actin filaments for 30 min at RT. Cell slides were mounted with DAPI Fluoromount-G (Southern Biotech) and examined using an AxioObserver Z.1 microscope (Carl Zeiss AG).

### Co-culture of lung fibroblasts and mature adipocytes

Mature adipocytes were differentiated as described above. 48 h after adipocytes seeding, primary human lung fibroblasts (IMR-90, Coriell Institute for medical Research, Camden, USA) were seeded in Corning™ Costar™ Transwell™ dishes (Thermo Fisher Scientific) and placed above the adipocytes containing medium. IMR-90 cells were tested negative for mycoplasma. The co-culture was subsequently cultured in adipocyte nutrition medium (Promocell). After additional 24 h, the lung fibroblast cavity was infected with IAV of an MOI of 1 in the same manner as described adipocyte section.

### Macrophage isolation, differentiation, and infection

Peripheral blood mononuclear cells were isolated from the peripheral blood of healthy donors under ethics approval No. 2019-1519_1 and differentiated as previously described [15].

Differentiated macrophages were infected with IAV (PR8) and the SARS-CoV-2 omicron and delta variants. Influenza infection was induced by incubating the cells with virus diluted in DPBS (Thermo Fisher Scientific) supplemented with 0.2% (v/v) human serum albumin (HSA; PAN-Biotech GmbH), 1 mM MgCl_2_, and 0.9 mM CaCl_2_ at 37 °C and 5% CO_2_ for 30 min. Cells were then incubated in RPMI medium supplemented with 1 mM MgCl_2_, 0.9 mM CaCl_2_, and 30 ng TPCK-treated trypsin (Thermo Fisher Scientific) for 8 h and 24 h. SARS-CoV-2 stocks were diluted in RPMI medium supplemented with 10% (v/v) HSA. Cells were incubated with inoculum for 60 min, washed once with DPBS, and infected for 8 h and 24 h.

### Protein quantification and western blotting

Cells were lysed in RIPA buffer (Thermo Fisher Scientific) and protein concentrations was determined with BCA-assay (Thermo Fisher Scientific). Proteins were subjected to SDS-PAGE and immunoblotted to polyvinylidene fluoride membranes (Thermo Fisher Scientific). Immunostaining was performed using primary antibodies against anti-ACE-2 (1:1,000; AF933, R&D Systems, Minneapolis, MN, USA) and anti-β-actin (1:1,000; A1978, Sigma Aldrich) and corresponding horseradish peroxidase (HRP)-conjugated anti-goat (HAF017, R&D Systems) or anti-mouse (172-1011, Bio-Rad Laboratories, Hercules, CA, USA) immunoglobulin G (IgG) secondary antibodies. Chemiluminescence was measured using Immobilon western chemiluminescent HRP substrate (Merck GmbH, Darmstadt, Germany) on a FusionFX imaging device (Vilber, Marne-la-Vallée, France).

### RNA extraction and qRT-PCR

RNA was extracted with the Qiagen RNeasy mini kit (Qiagen) and determined by the ND-1000 NanoDrop spectrophotometer (PEQLAB Biotechnologie GmbH, Erlangen, Germany) followed by a high-capacity cDNA reverse transcription kit (Thermo Fisher Scientific). qRT-PCR was performed using a Maxima SYBR Green qPCR master mix (Thermo Fisher Scientific) and 100 mM each forward and reverse primer pairs (Metabion International AG, Planegg, Germany) (Table S3). qRT-PCR cycle was run on a Rotor-Gene Q (Qiagen). The mRNA quantity was normalized to the *β*-actin housekeeping gene and all data were expressed as n-folds of the corresponding control. The ACE2 expression was normalized to the positive control Calu-3 cells.

Viral RNA was extracted using the QIAamp viral RNA mini kit (Qiagen). The qRT-PCR for IAV was performed using the RIDA^®^ GENE flu kit (R-Biopharm AG, Darmstadt, Germany) to detect the M-protein (Influenza A), NP (Influenza B), and H1 (IAV subtype H1N1v) genes. The SARS-CoV-2 copies were enumerated using the RIDA^®^ GENE SARS-CoV-2 kit (R-Biopharm AG) as previously described [14].

### Cytokine determination

Cytokines and chemokines in adipocyte and co-culture supernatants were measured with the LEGENDplex™ human adipokine panel (BioLegend, San Diego, CA, USA). The inflammatory responses in the macrophage supernatants were determined with the LEGENDplex™ COVID-19 cytokine storm panel 1 (BioLegend). Each specimen was analyzed with an Accuri C6 Plus cytometer (BD Biosciences, Franklin Lakes, NJ, USA) and the corresponding LEGENDplex v. 8.0 software (BioLegend).

### Histology and immunofluorescence

Tissue from in vivo mouse experiments and COVID-19 patient biopsies embedded in Tissue Tek O.C.T. compound was sliced into 4-µm sections with a Leica CM1950 microtome (Leica Microsystems, Wetzlar, Germany), mounted on Epredia^TM^ Superfrost^TM^ Plus adhesion microscope slides (Thermo Fisher Scientific), air-dried for 1 h, and stored at −20 °C. Mouse thoracic adipose were stained with hematoxylin-eosin (H&E) for the histological analyses.

Tissue cryosections were fixed in ice-cold acetone for 10 min for immunofluorescence staining. The tissues were blocked with 3% (v/v) BSA diluted in DPBS for 1 h. The primary antibodies used were rabbit anti-H1N1 influenza A virus nucleocapsid protein antibody (1:150; ab104870, Abcam, Cambridge, UK) and mouse anti-SARS-CoV-2 spike antibody (1:500; GTX632604, GeneTex, Irvine, CA, USA). After overnight incubation at 4 °C, sections were washed with PBS and incubated at RT for 45 min with the secondary antibodies Alexa Fluor^®^ 488 AffiniPure donkey anti-rabbit IgG (H + L; 1:500; 711-545-152, Jackson ImmunoResearch, West Grove, PA, USA) and Alexa Fluor^®^ 488 AffiniPure goat anti-mouse IgG (H + L; 1:500; 115-545-146, Jackson ImmunoResearch,) as well as BODIPY™ 558/568 phalloidin (1:500; B3475, Thermo Fisher Scientific). The sections were mounted with 4',6-diamidino-2-phenylindole (DAPI) Fluoromount-G (Southern Biotech, Birmingham, AL, USA) and imaged with AxioObserver Z.1+Apotome 2 (Carl Zeiss AG, Oberkochen, Germany).

### Immunocytochemistry

Cells were fixed in 4% (v/v) paraformaldehyde (PFA) in PBS at 37 °C for 30 min, permeabilized with PBS containing 0.1% (w/v) Triton-X and blocked with 3% (v/v) BSA in PBS at RT for 30 min. Adipocyte cytoplasm was stained with wheat germ agglutinin conjugated with Alexa Fluor^®^ 555 (W32464, Thermo Fisher Scientific) according to the manufacturer’s instruction before fixation. Adipocytes and macrophages were both incubated with mouse anti-dsRNA monoclonal antibody J2 (1:500; RNT-SCI-10010200, Jena Bioscience, Jena, Germany) or rabbit anti-dsRNA monoclonal antibody J2 (1:500, Kf-Ab01299-23.0, kerafast, Boston, MA, USA) to detect viral RNA. Additionally, macrophages were stained with mouse anti-CD68 monoclonal antibody (1:200; 14-0688-82, Invitrogen). Both primary antibodies were diluted in 3% (v/v) BSA in PBS incubated at 4 °C overnight. Secondary antibodies Alexa Fluor^®^ 488-conjugated donkey anti-rabbit (711-545-152, Jackson ImmunoResearch) and Cy-3-conjugated donkey anti-mouse (715-165-150, Jackson ImmunoResearch) were each applied at 1:500 dilution. Cells were mounted with DAPI Fluoromount-G (Southern Biotech) and examined with an AxioObserver Z.1 microscope (Carl Zeiss AG).

### Statistical analyses and scheme design

Statistical analyses was performed using GraphPad Prism v. 9 (GraphPad Software, La Jolla, CA, USA). Individual values are presented either as violin plots with the median indicated by a dashed line or as bar charts displaying the mean ± standard deviation. Detailed descriptions of all methods and measures can be found in the corresponding figure legends. The exact *p* values are reported in the main text for clarity and reference. All schematic illustrations were created with BioRender (https://biorender.com).

## Results

### Obesity is associated with disease progression and high viral loads in influenza virus-infected mice

**Fig. 1:**
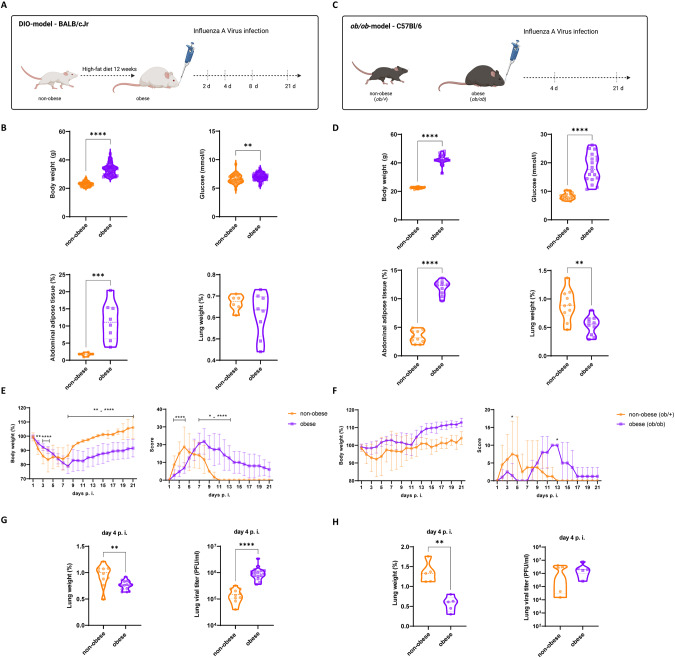
Obesity is associated with disease progression and high viral loads in influenza virus-infected mice. **A** Schematic overview of non-obese and diet-induced obese (DIO), female BALB/C mice with subsequent infection with IAV. **B** DIO mice (*n* = 64) were characterized by increased body weight and blood glucose, compared non-obese (*n* = 46). Also, relative mass of adipose tissue extracted from the abdomen were elevated, and relative lung weights decreased in obese (*n* = 8) compared to non-obese (*n* = 6). **C** Schematic overview of female C57BL/6 non-obese, heterozygous (ob/+), and homozygous obese mice with global leptin knockout (*ob/ob*) with subsequent infection with IAV. **D** obese *ob/ob* mice (*n* = 20) were characterized by increased body weigh tand blood glucose compared to non-obese *ob/+* mice (*n* = 20). Also, relative mass of adipose tissue was elevated and relative lung weights decreased in *ob/ob* (*n* = 10) compared to non-obese *ob/+* (*n* = 10*)* mice. **E** Relative body weight and overall scoring values after infection of DIO showed a significant temporal right shift and a slower recovery after 21 days p.i. **F** Infection of *ob/ob* mice led to lesser alteration in the body weight progression but equal overall burden score progression compared to DIO. Relative lung weights were decreased in obese mice of DIO (*n* = 16) compared to non-obese (*n* = 10) (**G**) and *ob/ob* (*n* = 5) (**H**) compared to *ob/+* (*n* = 5) mice day 4 p.i. Active virus particles determined via plaque assay of lung homogenates were significantly increased in DIO mice (**G**), and elevated in *ob/ob* mice (**H**). **P < 0.01, ***P < 0.001, ****P < 0.0001. P calculated by Mann–Whitney-test (**B**, **D**, **G**, **H**) and Two-way ANOVA, Šidáks multiple comparisons (**E**, **F**), *P < 0.05, **P < 0.01, ****P < 0.0001.

We induced IAV infection in two mouse models to investigate the impact of obesity on respiratory viruses (Fig. 1A–H). For this, a female BALB/c mouse DIO model was used to simulate an obesity-promoting western diet in humans (Fig. 1A). The mice in the DIO group presented with significant weight gain (*p* < 0.0001) and elevated blood glucose levels (*p* = 0.0015) compared to the control mice (Fig. 1B). A massive gain in adipose tissue largely accounted for the observed 50% increase in body weight. The adipose tissue was visible during tissue sectioning and was quantified after calculating the amount of abdominal adipose tissue extracted (*p* = 0.0007) (Fig. 1B). By contrast, the lung weight was relatively lower in the obese than the control mice (Fig. 1B).

To confirm our findings independently in another genetic background, we also analyzed IAV infection in the monogenic *ob/ob* obesity model characterized by global leptin knockout (KO) (Fig. 1C) [16, 17]. To this end, we used C57Bl/6 mice which differed from the BALB/c mainly in terms of their innate immune response [18]. The leptin KO (*ob/ob* mutant) presented with an obese phenotype as well as corresponding metabolic alterations such as insulin resistance and hyperglycemia [19]. The *ob/ob* mice were characterized by elevated body weight compared to non-obese control mice (*p* < 0.0001) (Fig. 1D). The blood glucose levels of the *ob/ob* mice were significantly higher than those of the control (*p* < 0.0001) (Fig. 1D). The total adipose tissue weight was significantly higher (*p* < 0.0001) but the lung weight was substantially lower (*p* = 0.0042) in the obese than the non-obese mice (Fig. 1D).

IAV pandemic H1N1 strain 2009 (pdmH1N1) was intranasally administered to the DIO- and *ob/ob* mouse models. The infection course was analyzed for 21 days using a predefined scoring system to evaluate disease severity based upon categories, observations, and corresponding gradings (Table S1, Fig. 1E, F).

In the DIO model, the non-obese mice exhibited maximum weight loss 4 days post-infection (p.i.). By contrast, the obese mice had lost only ≤~20% of their original body weight and the weight loss peaked on day 8 (Fig. 1E). Unlike the non-obese mice, the obese mice did not recover to the control standard score until day 21. The obese mouse scores reflected a rightward shift in the infection curve and the inability to recover fully from the infection (Fig. 1E).

We compared the p.i. body weights of the *ob/ob* and the heterozygote, non-obese control (*ob/+*) mice and found that symptom onset was delayed in the former (Fig. 1F). The scores demonstrated delayed but aggravated disease progression as seen with the DIO model (Fig. 1F).

The lungs of the infected obese DIO (*p* = 0.0035) and *ob/ob* (*p* = 0.0079) mice weighed less than those of the infected non-obese mice. By day 4, however, the viral titers were significantly higher in the infected DIO mice (*p* < 0.0001) (Fig. 1G). Virus titers in *ob/ob* mice showed the same tendency (Fig. 1H).

### Thoracic adipose tissue adjacent to the lungs harbored influenza virus

**Fig. 2:**
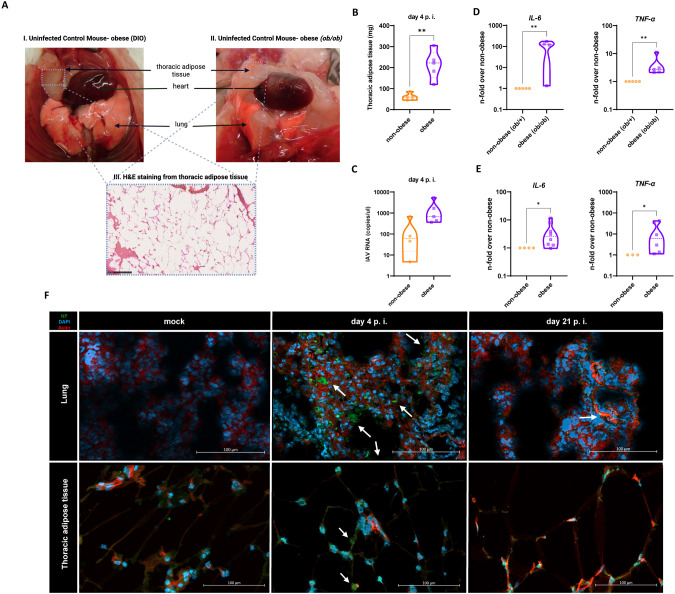
Thoracic adipose tissue adjacent to the lungs harbored influenza virus. **A** Image of the thorax lumen of uninfected DIO (left) and *ob/ob* mice (right) with H&E staining of a representative cryosection (4 µm thickness) of the extracted thoracic adipose tissue, Scale bar, 250 μm. **B**, **C** *ob/ob* (*n* = 5) mice showed increased thoracic adipose tissue mass (**B**) and elevated copies/mg of IAV within the extracted tissue day 4 p.i. compared to non-obese (*n* = 5). Extracted abdominal adipose tissue sections from obese *ob/ob* (**D**) and DIO mice (**E**) show upregulated mRNA levels of IL-6 and TNF-α compared to the corresponding non-obese mice. **F** IAV nucleoprotein (green) visualized by immunofluorescence staining detected at day 4 p.i. (arrowhead) within cryosections (4 µm) from lung and additionally extracted thoracic adipose tissue from representative *ob/ob* mice. Phalloidin (red) and DAPI (blue) staining were used to detect actin and the nuclei. Scale bar 100 μm. *P* calculated by Mann-Whitney-test (**B**, **D**, **E**). *P < 0.05**P < 0.01.

Investigation of the thoracic lumen of DIO and *ob/ob* mice revealed abundant adipose tissue masses between the lungs, heart, and pleura in the mediastinum (Fig. 2A). The extensive accumulation of thoracic adipose tissue and its proximity to the lungs were indicative of the close relationship between lung and fat tissue. Macroscopic images of the thoracic situs demonstrated the proximity between the lung and adipose tissue (Fig. 2A).

The *o*b*/ob* mice presented with significantly more thoracic adipose tissue adjacent to the lungs than the *ob/+* mice 4 day p.i. (*p* = 0.0079) (Fig. 2B). In obese mice, then, elevated viral RNA could be correlated with simultaneous increases in thoracic adipose tissue (Fig. 2C), yet reaching no significant level (*p* = 0.0794)

We measured the interleukin (IL)-6 and tumor necrosis factor alpha (TNF-α) expression levels in the adipose tissue of the mice. The IL-6 (*p* = 0.0108) and TNF-α levels (*p* = 0.0075) were higher in the adipose tissue of the obese (*ob/ob*) than the non-obese (*ob/+*) mice (Fig. 2C). This was consistent in the DIO model (Fig. 2D, IL-6 (*p* = 0.0444), TNF- α (*p* = 0.0238)). Moreover, in the thoracic adipose tissue, we detected significantly elevated TNF-α levels in *ob/ob* mice (*p* = 0.0286, Fig. S2A) and elevated IL-6 levels in DIO mice (*p* = 0.0079, Fig. S2B). We also prepared cryosections of the left lung lobes and thoracic adipose tissue of the infected mice. Immunofluorescence staining of the tissue sections revealed positive signal for the viral protein in the lungs on day 4 and even on day 21 in the obese *ob/ob* mice (Fig. 2D, upper panel). Influenza virus was also detected in the thoracic adipose tissue of the same mice (Fig. 2D, lower panel).

### Influenza virus replicated in human primary adipocytes

**Fig. 3:**
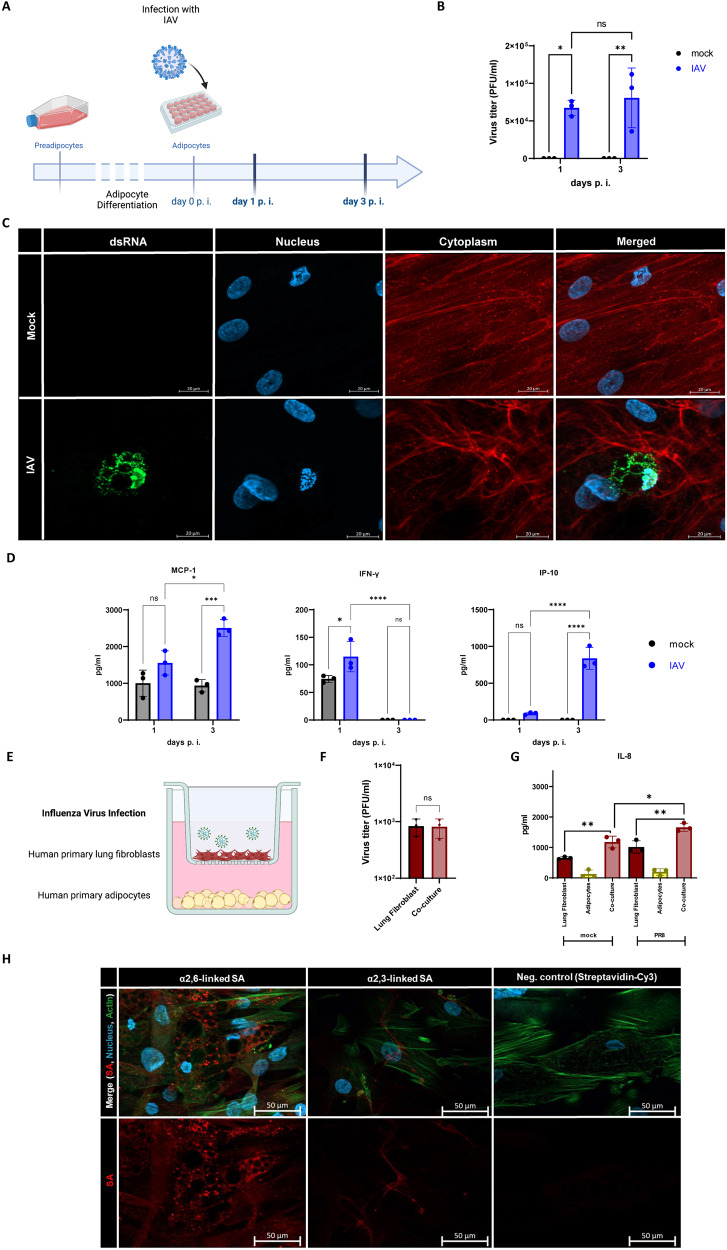
Influenza virus replicated in human primary adipocytes. **A** Schematic overview of the experimental setup for the infection of primary derived adipocytes differentiated from preadipocytes and the subsequent infection with IAV (PR8) for 1 and 3 days with an MOI (Multiplicity of infection) of 1. **B** Active virus particles detected by standard plaque assay from supernatants of infected adipocytes were significantly increased after 1 and 3 days of infection with PR8 with an MOI of 1. **C** dsRNA (green) of IAV could be visualized by immune fluorescence staining within infected adipocytes day 3 p.i., mainly located at the lipid droplets and already damaged nucleus within the cells. Cytoplasm (red) was stained wheat germ agglutinin (WGA) and the nuclei (blue) with DAPI. Scale bars, 20 μm. **D** Infected adipocytes secreted significantly more IFN-γ, 1 day p.i. and more MCP-1, IP-10 3 days p.i. **E** Schematic illustration of the infection of primary lung fibroblasts co-cultivated in a trans-well system with adipocytes with IAV of an MOI of 1. **F** No differences in virus titer were determined via plaque assay in the co-culture compared to the mono-culture of solely lung fibroblasts. **G** Co-culture of adipocytes with lung fibroblasts led to a significant upregulation of IL-8 in the supernatants at 24 h p.i. **H** Presence of α-2,6-linked (SNA) and α-2,3 linked (MAL II) SA residues in differentiated human adipocytes stained with biotinylated lectins and the corresponding fluophore-linked streptavidin. Actin filaments were stained with phalloidin (green) and the nuclei (blue) with DAPI. Scale bars, 50 µm. Data presented as Mean ± SD and presentative of three independent biological replicates; *P* calculated by two-way analysis of variance (ANOVA) test, Šidáks multiple comparisons test (**B**), Tukeys’ multiple comparisons test (**D**); Mann-Whitney test (**F**), and Ordinary one-way ANOVA (**G**); *P < 0.05, **P < 0.01, ***P < 0.001, ****P < 0.0001.

We differentiated primary human preadipocytes to mature adipocytes (Fig. 3A). The maturation process was monitored by observing intracellular lipid droplet development and the loss of the fibroblast morphology of the cells (Fig. S1). Subsequently, we infected the mature adipocytes with influenza virus A/Puerto Rico/8/34 (IAV/H1N1/PR8) and measured the viral titers after 1 day and 3 days.

Viral titers were significantly higher in the IAV-infected adipocytes compared to mock. Hence, positive viral replication had occurred on days 1 and 3 p.i. (Fig. 3B). Immunofluorescence staining of double-stranded (ds)RNA occurs exclusively during single-stranded (ss)RNA virus replication. Clear signals were detected on day 3 in the adipocytes infected with IAV (Fig. 3C). Moreover, significant interferon gamma (IFN-γ) upregulation in the cellular supernatants at 24 h p.i. (*p* = 0.0329) could be observed. Monocyte chemoattractant protein (MCP) 1 (*p* = 0.0006) and IFN-γ-induced protein (IP)-10 (*p* = 0.0079) were significantly upregulated after 3 days p.i. (Fig. 3D).

The adipocyte/human primary lung fibroblast co-culture was used to assess the impact of inflammatory active adipocytes on influenza virus replication. We infected co-cultivated lung fibroblasts with IAV for 24 h and evaluated the influence of inflammatory active human primary adipocytes in a transwell system (Fig. 3E). The viral titers did not significantly differ between the single-cultured fibroblasts and the co-culture system (Fig. 3F). Nevertheless, we observed synergy between the co-cultured cells. Significant upregulation of the proinflammatory cytokine IL-8 was detected in the infected co-culture compared to non-infected cells (*p* < 0.0153) (Fig. 3G). In addition, we also quantified levels of IL-6 and MCP-1 in the supernatants. Although there was a slight upward trend observed in infected lung fibroblast, we did not observe a significant difference compared to the co-cultured cells (Fig. S2).

During the cell entry of influenza A viruses, the hemagglutinin molecules in the virus envelope recognize terminal SA residues with an α-2,3 and α-2,6 linkage of surface glycoconjugates [20]. In order to confirm the active replication of IAV in mature adipocytes, we performed additional fluorescent staining to visualize the presence of SA residues. Notably, the cells exhibited a prominent signal for α-2,6-linked SA in close proximity to intracellular lipid droplets, while the detection of α-2,3-linked SA was observed to a lesser extent (Fig. 3H).

### SARS-CoV-2 was detected in the thoracic adipose tissue of COVID-19 patients

**Fig. 4:**
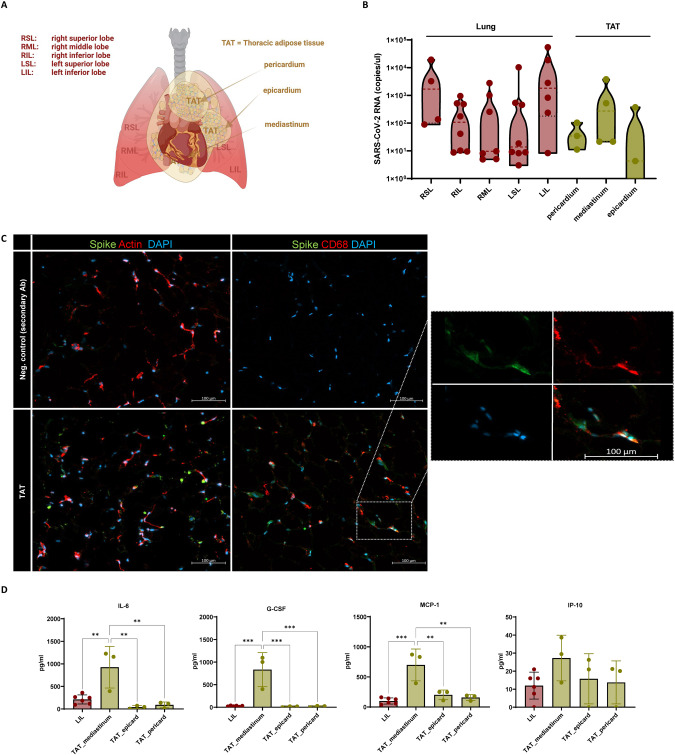
SARS-CoV-2 was detected in the thoracic adipose tissue of COVID-19 patients. **A** Schematic overview of the localization of the lung and adipose tissues biopsies taken from the thorax of five deceased COVID patients. **B** SARS-CoV-2 RNA was detected within different areas of the lung and in extracted thoracic adipose tissue (TAT) samples. **C** Virus particles were detected in cryosections from TAT (mediastinum) determined by immunofluorescence staining of the spike protein (green), actin (phalloidin, red) and nuclei (DAPI, blue) compared to the corresponding negative control (upper panel). Additional staining of CD68 from macrophages revealed the potential colocalization of macrophages and spike protein within TAT (mediastinum). Scale bar, 100 µm (lower panel). **D** Proinflammatory markers IL-6, G-CSF, MCP-1, and IP-10 were higher in homogenized tissue from TAT (mediastinum) compared to biopsies from the left inferior lung lobe (LIL). Data presented as Mean ± SD. *P* calculated by Ordinary one-way analysis of variance (ANOVA), multiple comparisons; **P < 0.01, ***P < 0.001.

We examined biopsies from five deceased COVID-19 patients and analyzed various lung areas and the thoracic adipose tissues (Fig. 4A). In all areas, SARS-CoV-2 omicron variant could be confirmed by measuring SARS-CoV-2 RNA concentration (Fig. 4A, B). The highest amount of viral RNA was detected in the left lower lung lobes (Fig. 4B). However, high concentrations were also found in the mediastinal adipose tissue (Fig. 4B) and immunofluorescence staining revealed SARS-CoV-2 spike protein there (Fig. 4C). Immunofluorescence staining disclosed potential macrophage/SARS-CoV-2 spike protein colocalization (Fig. 4C).

The thoracic and especially the mediastinal adipose tissue were in a highly active inflammatory state as proinflammatory factors were detected in them. The mediastinal adipose tissue exhibited high SARS-CoV-2 loads (Fig. 4D). In addition, IL-6 (*p* = 0.0026), granulocyte-colony stimulating factor (G-CSF) (*p* = 0.0001), and MCP-1 (*p* = 0.0001) were significantly upregulated in the mediastinal adipose tissue compared to the examined lung tissue. IP-10 levels were additionally upregulated yet reaching no significant level.

### SARS-CoV-2 variants did not replicate in human primary adipocytes

**Fig. 5:**
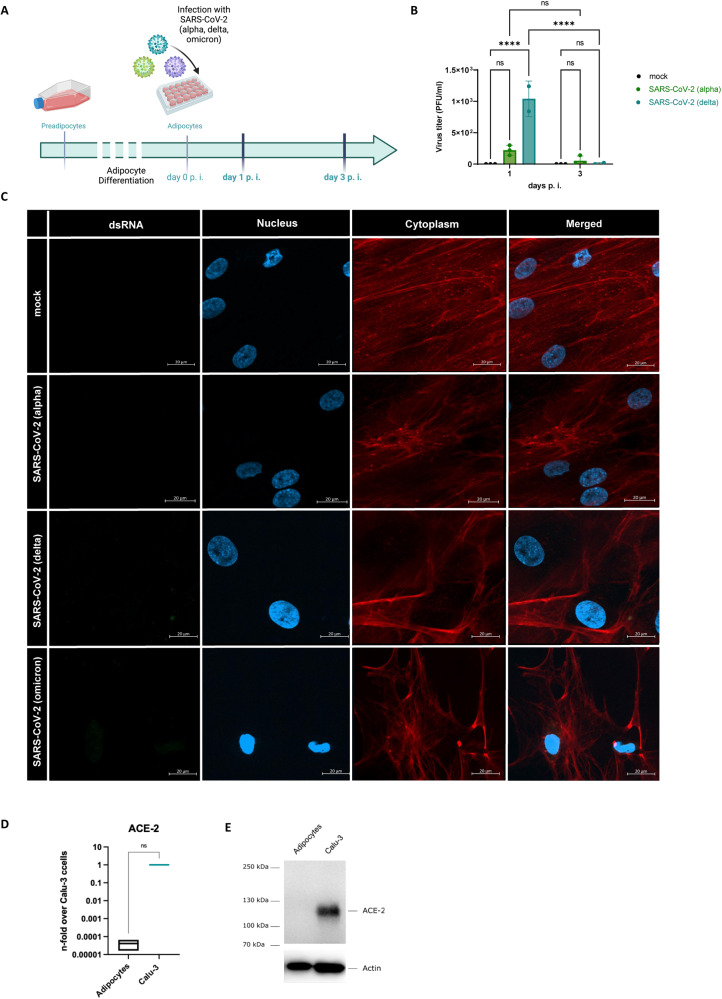
SARS-CoV-2 variants did not replicate in human primary adipocytes. **A** Schematic overview of the experimental setup for the infection of primary derived adipocytes differentiated from preadipocytes and the subsequent infection with the SARS-CoV-2 variants alpha, delta, and omicron for 1 and 3 days. **B** Active virus particles of the alpha and delta variant were detected 1 day p.i. but decreased until day 3 p.i. **C** dsRNA (green) of SARS-CoV-2 of all three variants were not visible within the infected adipocytes after day 3 determined by immunofluorescent staining of infected cells compared to uninfected mock cells. **D** mRNA expression of was not detected in human primary adipocytes compared to Calu-3 cells as positive control. **E** Western blot analysis of the ACE-2 protein revealed no expression in adipocytes compared to the Calu-3 positive control. Actin served as loading control. Data presented as Mean ± SD and presentative of two independent experiments for each variant; *P* calculated by two-way analysis of variance (ANOVA) test, Tukey’s multiple comparisons test (**B**) and Mann–Whitney test (**D**); ****P < 0.0001.

To demonstrate the ability of SARS-CoV-2 to replicate in adipose tissue, we infected human primary adipocytes with the SARS-CoV-2 alpha, delta, and omicron variants for 24 h and 72 h (Fig. 5A). Viral particles were detected on day 1 p.i. Nevertheless, the levels of the SARS-CoV-2 delta variant had significantly declined after 3 days (*p* < 0.0001). This decline was also seen for the alpha variant (Fig. 5C).

The omicron variant titer could not determine by plaque assay, due to the inability to induce a strong cytopathic effect [21, 22]. Hence, we did not perform the plaque assay but evaluated the dsRNA for all variants by immunofluorescence staining and could not detect dsRNA within the cells (Fig. 5C).

Additionally, Angiotensin-converting enzyme 2 (ACE-2) receptor mRNA in adipocytes was analyzed. Compared to Calu-3 cells, used as a positive control, no mRNA expression was detectable (*p* = 0.05). Moreover, no ACE-2 protein was determined in adipocytes (Fig. 5D, E).

### Macrophages contributed to inflammation even in the absence of active viral replication

**Fig. 6:**
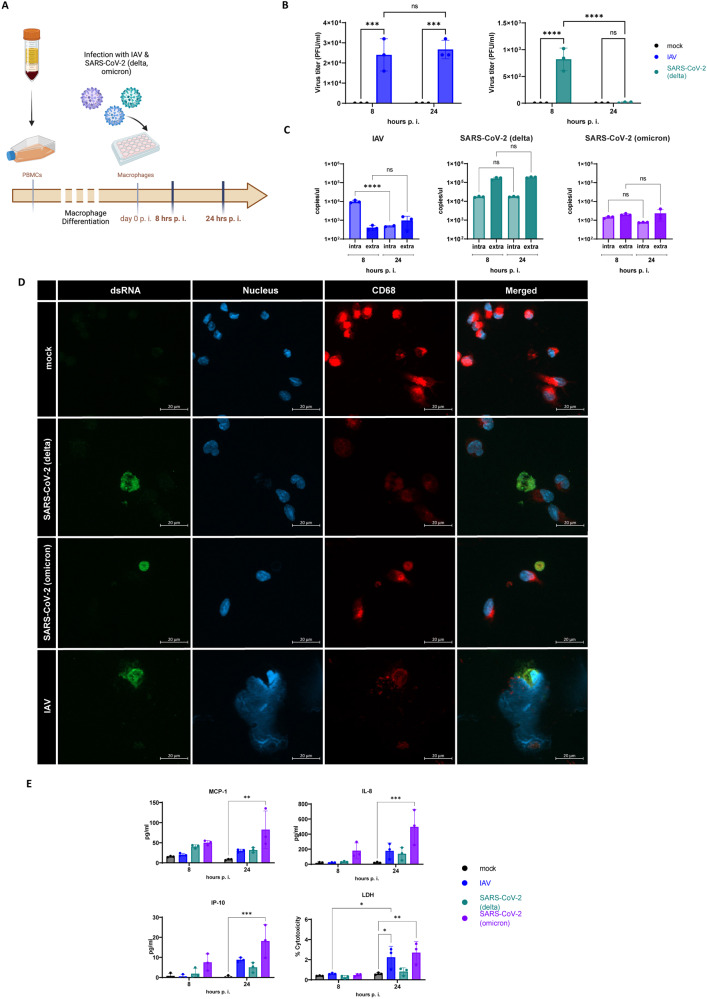
Macrophages contributed to inflammation even in the absence of active viral replication. **A** Schematic overview of the experimental setup for the infection of macrophages derived from isolated PBMCs from a healthy donor with subsequent infection with IAV and SARS-CoV-2 (delta and omicron) for 8 and 24 h. **B** Active virus particles of IAV were detected after 8 and 24 h, whereas SARS-CoV-2 (delta) particles decreased from 8 to 24 h. **C** Viral RNA was determined extracellular (extra) and intracellular (intra) of infected macrophages. **D** dsRNA (green) of both SARS-CoV-2 and IAV detected by immune fluorescence staining within PBMCs stained with CD68 (red) and DAPI (blue) 24 h p.i. Scale bars 20 µm. **E** Proinflammatory markers MCP-1, IL-8, and IP-10 were significantly upregulated in supernatants of macrophages infected with the omicron variant. SARS-CoV-2 (omicron) and IAV led to increased relative cytotoxicity measured by Lactate dehydrogenase (LDH)-assay in cell supernatants 24 h p.i. Data presented as Mean ± SD and presentative of three independent experiments for each variant; *P* calculated by two-way analysis of variance (ANOVA) test, Tukey’s multiple comparisons test (**B**, **E**), Ordinary one-way ANOVA, Tukey’s multiple comparisons (**C**), *P < 0.05, **P < 0.01, ***P < 0.001, ****P < 0.0001.

Immunofluorescence staining of the adipose tissue from deceased COVID-19 patients revealed SARS-CoV-2 spike protein colocalization with the macrophage marker CD68 (Fig. 4C). To explore the possibility that IAV and SARS-CoV-2 replicate inside macrophages, we examined human monocyte-derived macrophages at 8 h and 24 h p.i. (Fig. 6A).

We identified active IAV and SARS-CoV-2 (delta) particles at 8 h p.i. However, only IAV could still be detected after 24 h p.i. albeit at a lower level than at 8 h p.i. (Fig. 6B). We additionally measured the intracellular and extracellular RNA levels of all virus variants. Neither concentration increased over time (Fig. 6C). On the other hand, immunofluorescence staining disclosed IAV and SARS-CoV-2 dsRNA within the macrophages (Fig. 6D).

The macrophages infected with the omicron variant presented an increase in proinflammatory response. Here, MCP-1 (*p* = 0.0012), IL-8 (*p* = 0.0007), and IP-10 (*p* = 0.0003) levels 24 h p.i. could be identified (Fig. 6E). A lactate dehydrogenase assay showed significantly elevated macrophage damage in response to IAV (*p* = 0.0424) and SARS-CoV-2 omicron variant (*p* = 0.0063) infection (Fig. 6E).

## Discussion

The association between obesity and severe respiratory infections first became evident during the 2009 influenza virus pandemic and once again during the more recent COVID-19 pandemic. To identify the pathophysiological factors linking obesity and viral respiratory infections, we used two distinct murine obesity models for influenza virus infection and completed our findings by using human-specific primary cells. To investigate the interaction of adipose tissue with further RNA-viruses, we analyzed deceased COVID-19 patients and performed SARS-CoV-2 in vitro experiments.

In this study, we investigated whether various obesity-related mechanisms contribute to the progression of respiratory infections. By using two distinct mouse obesity models for influenza virus infection, an increase of infection severity accompanied with a protracted disease progression with elevated viral titer in obese animals could be clearly demonstrated. These results were corroborated by other studies showing overall increased mortality in obese DIO mice infected with a pdmH1N1 strain [23–26]. However, to the best of our knowledge, the present study was the first to demonstrate a prolonged course of influenza virus infection in obese animals (Fig. 1).

Interestingly, we detected influenza virus particles in the thoracic adipose tissue adjacent to the lungs and confirmed a replication in the adipose tissue near the lungs (Fig. 2). In this context, previous studies have reported the detection of virus particles in different parts of omental adipose tissue in mice infected with an avian influenza subtype (H5N1) [27]. Hence, the massive lipid expansion that occurs in patients with obesity provides a viral replication site in close proximity to the lungs that may directly affect the course of viral infection. This finding may provide a potential explanation for the observed increased shedding of IAV in symptomatic patients with obesity [28]. The infection of human primary adipocytes with IAV clearly identified a possible virus replication (Fig. 3). This finding is consistent with previously published studies positive influenza signals in differentiated IAV and as well as other pathogens, such as CMV or Adenovirus [29]. However, conditioned media of the adipocytes had no effect on viral replication in primary lung fibroblasts in a co-culture system (Fig. 3E). One limitation of this study is the use of a human influenza isolate for the in vitro studies and the mouse-adapted strain for the in vivo infection models, even though both strains belong to the same H1N1 subtype.

Nevertheless, our results demonstrated that proinflammatory factors and inflammatory activity were highly upregulated in abdominal adipose tissue of *ob/ob* mice. These findings corroborated those of prior studies. An earlier work indicated that adipose tissue had high proinflammatory activity in obesity and suggested that the low-grade inflammation associated with obesity is critical in the course of pneumonia [10].

In order to transfer our findings to other RNA viruses, we analyzed the thoracic adipose tissue of five deceased COVID-19 patients with obesity (Fig. 4). All patients were infected with the omicron variant of SARS-CoV-2. Our findings clearly showed that SARS-CoV-2 RNA could be detected in the adipose tissue adjacent to the lung. The mediastinal/periaortic adipose tissue showed very high concentrations of SARS-CoV-2 RNA and proinflammatory proteins, aligning with the concepts previously summarized by Ryan and Caplice that the dissemination of virus particles to adipose tissue and subsequent pro-inflammatory response could be a contributing factor to the increased risk observed in individuals with obesity for COVID-19 [30]. However, we could neither detect ACE-2 mRNA nor protein in our in vitro experiments by using adipocytes (Fig. 5E, F). It has been previously reported that adipose tissue, particularly during obesity, serves as a potential reservoir for SARS-CoV-2 due to the upregulation of ACE-2 mRNA [31]. Based on our results, we cannot identify adipocytes as the primary site of SARS-CoV-2 replication in connection to the obesity risk factor.

This lack of ACE-2 receptor protein is consistent with the detected decline of viral titers of different SARS-CoV-2 variants in human primary adipocytes. Moreover, immunofluorescence staining revealed no dsRNA (Fig. 5). In contrast, Zickler et al. reported active replication of SARS-CoV-2 in adipocytes and intriguingly used IAV as negative control for the infection [32]. However, it is important to note that adipose tissue consists not only of adipocytes but also of macrophages [33]. Therefore, the presence of SARS-CoV-2 RNA in the thoracic adipose tissue of deceased patients may originate co-localization of spike protein with macrophages (Fig. 4). Hence, we infected human monocyte-derived macrophages with SARS-CoV-2. Interestingly, omicron variant was detected in the macrophages and an upregulation of proinflammatory factors could observed (Fig. 6).

The present study demonstrated a direct association between obesity and pneumonia by showing that IAV infects adipocytes which may, in turn, serve as reservoirs for replicating viral particles. However, the presence of SARS-CoV-2 omicron variant in thoracic adipose tissue, might be mainly explained by the macrophages within the tissue and not the adipocytes.

Our data demonstrate that certain respiratory viruses replicate in human primary adipocytes. This discovery identified a novel source for respiratory virus that contributes to the viral load within the thorax. Moreover, our findings revealed that thoracic adipose tissue is an important source for respiratory viruses, functions as a niche for virus particles, and might account for the prolonged course of certain respiratory infections. Future research should, therefore, endeavor to design, optimize, and validate innovative therapeutic strategies that eliminate the viral reservoirs in thoracic adipose tissue.

### Supplementary information

Supplemental Legends

Supplemental Figure S1

Supplemental Figure S2

Supplemental Figure S3

Supplemental Table S1

Supplemental Table S2

Supplemental Table S3

## Data Availability

The data presented in the manuscript have not yet been made available.
