# Supplementary material for: Thoracic adipose tissue contributes to severe virus infection of the lung
Source: Int J Obes (Lond). 2023 Aug 16;47(11):1088–99. doi: 10.1038/s41366-023-01362-w (PMC10599992; doi:10.1038/s41366-023-01362-w)
Supplement: Supplementary file 1 — Supplemental Legends [file 41366_2023_1362_MOESM1_ESM.docx]

Supplementary Figure S1. Schematic overview of experimental steps for the differentiation of human preadipocytes to mature adipocytes according to manufacturers’ instructions by PromoCell with representative microscopical images. At first, preadipocytes were cultivated until reaching 100 % confluency. Afterward, medium was changed to preadipocyte differentiation medium for 72 h. Finally, cells were cultured in adipocyte nutrition medium until sufficient development of intracellular lipid droplets as a sign of maturity.

Supplementary Figure S2. Expression of IL-6 and TNF- α in thoracic adipose tissue from obese mice. Extracted thoracic adipose tissue sections from obese *ob/ob* (A) and DIO mice (B) show upregulated mRNA levels of IL-6 and TNF-α compared to the corresponding non-obese mice. P calculated by Mann-Whitney-test (B, D, E). *P<0.05**P<0.01.

Supplementary Figure S3. Levels of IL-6 and MCP-1 of co-culture of adipocytes with lung fibroblasts at 24 h p. i.. Factors were determined in supernatants of all three settings: Lung fibroblasts, adipocytes, and the corresponding co-culture with and without infection with the IAV PR8 strain. Data presented as Mean ± SD and presentative of three independent experiments.
